# HSV Neutralization by the Microbicidal Candidate C5A

**DOI:** 10.1371/journal.pone.0018917

**Published:** 2011-05-06

**Authors:** Lot de Witte, Michael D. Bobardt, Udayan Chatterji, Freek B. van Loenen, Georges M. G. M. Verjans, Teunis B. H. Geijtenbeek, Philippe A. Gallay

**Affiliations:** 1 The Center for Experimental and Molecular Medicine, Academic Medical Center, University of Amsterdam, Amsterdam, The Netherlands; 2 Department of Immunology and Microbial Science, The Scripps Research Institute, La Jolla, California, United States of America; 3 Department of Virology, Erasmus Medical Centre, Rotterdam, The Netherlands; INSERM, France

## Abstract

Genital herpes is a major risk factor in acquiring human immunodeficiency virus type-1 (HIV-1) infection and is caused by both Herpes Simplex virus type 1 (HSV-1) and HSV-2. The amphipathic peptide C5A, derived from the non-structural hepatitis C virus (HCV) protein 5A, was shown to prevent HIV-1 infection but neither influenza nor vesicular stomatitis virus infections. Here we investigated the antiviral function of C5A on HSV infections. C5A efficiently inhibited both HSV-1 and HSV-2 infection in epithelial cells *in vitro* as well as in an *ex vivo* epidermal infection model. C5A destabilized the integrity of the viral HSV membrane. Furthermore, drug resistant HSV strains were inhibited by this peptide. Notably, C5A-mediated neutralization of HSV-1 prevented HIV-1 transmission. An *in vitro* HIV-1 transmigration assay was developed using primary genital epithelial cells and HSV infection increased HIV-1 transmigration. Treatment with C5A abolished HIV-1 transmigration by preventing HSV infection and by preserving the integrity of the genital epithelium that was severely compromised by HSV infection. In conclusion, this study demonstrates that C5A represents a multipurpose microbicide candidate, which neutralizes both HIV-1 and HSV, and which may interfere with HIV-1 transmission through the genital epithelium.

## Introduction

Genital herpes is the other most prevalent sexually transmitted infections worldwide and is the most common cause of genital ulcers. Genital herpes is mainly caused by HSV-2, although an increasing percentage of the genital herpes is caused by HSV-1 [1]–[3]. The hallmark of herpesvirus infections is the establishment of a lifelong, latent infection that can reactivate to cause one or more rounds of disease. In the USA, 40 to 60 million people are HSV-2-infected, with an incidence of 1–2 million new infections and 600,000–800,000 clinical cases per year [3]. Prevalence in the 30–40 year-old population is about 30% [3]. There is a significant medical need for prevention and treatment of HSV-2 since there are no licensed vaccines currently available and therapeutic treatment requires repeated dosing with antiviral products. Importantly, genital herpes is a risk factor to acquire HIV-1 infection by sexual contact, by increasing both infectivity and susceptibility to acquire HIV-1 [1]–[3]. Genital herpes is characterized by the formation of papules and vesicles, which can progress into pustules and ulcers. Ulceration could disrupt the mucosal barrier and thereby abrogated the protective barrier function of the epithelium. Moreover, ulceration could allow HIV-1 to reach the sub-epithelial dendritic cells (DC), which efficiently promotes HIV-1 transmission *in vitro* [4]. Furthermore, target cells for HIV-1 are attracted to the mucosal sites during HSV-2 infection [4]–[6], which can result in higher transmission rates. Thus, there is also an urgent need for novel prophylactic methods, such as topical microbicides designed for genital application, to prevent both HSV and HIV-1 transmission. Development of topical microbicide with dual activity that target both HIV-1 and HSV may prove a powerful strategy for reducing HIV-1 as epidemiological studies consistently demonstrate synergy between these two pathogens.

The short peptide called C5A derived from HCV nonstructural protein 5A (NS5A) has antiviral activity against HCV and HIV-1 [7]–[8]. Importantly, C5A represents a novel class of microbicidal candidates against HIV-1. C5A neutralizes primary HIV-1 and SIV isolates in nM-µM concentrations without apparent cytotoxicity to human cells [8]. C5A corresponds to a small (18 amino acids) N-terminal region (aa 3–20) NS5A (477 amino acids) [7]. The sequence of C5A encompasses the region responsible for the anchoring of NS5A into the ER membrane [9]. In contrast to C5A (18 amino acids), full-length NS5A (477 amino acids) does not inhibit HIV-1 infection [8]. We demonstrated that C5A disrupts HIV-1, but preserves the integrity of the cellular plasma membrane [8]. The HIV-1 membrane rupture by C5A is apparently virus-specific because it does not inhibit the infection of other enveloped viruses, such as influenza and vesicular stomatitis viruses [8]. It is unclear whether C5A can prevent co-infections such as HSV that enhance HIV-1 susceptibility.

Here we have investigated the antiviral activity of C5A against HSV-1 and HSV-2. Our data demonstrate that C5A not only prevents HSV infection but also limits viral dissemination. Furthermore, the study demonstrates that C5A prevents HSV-induced HIV-1 susceptibility. Thus, our data show that C5A is an efficient antiviral peptide that prevents HSV as well as HIV-1 infection. This function might be harnessed in microbicides that need to prevent HIV-1 transmission.

## Results

### C5A inhibits HSV-1 and HSV-2 infection *in vitro*

**Figure 1:**
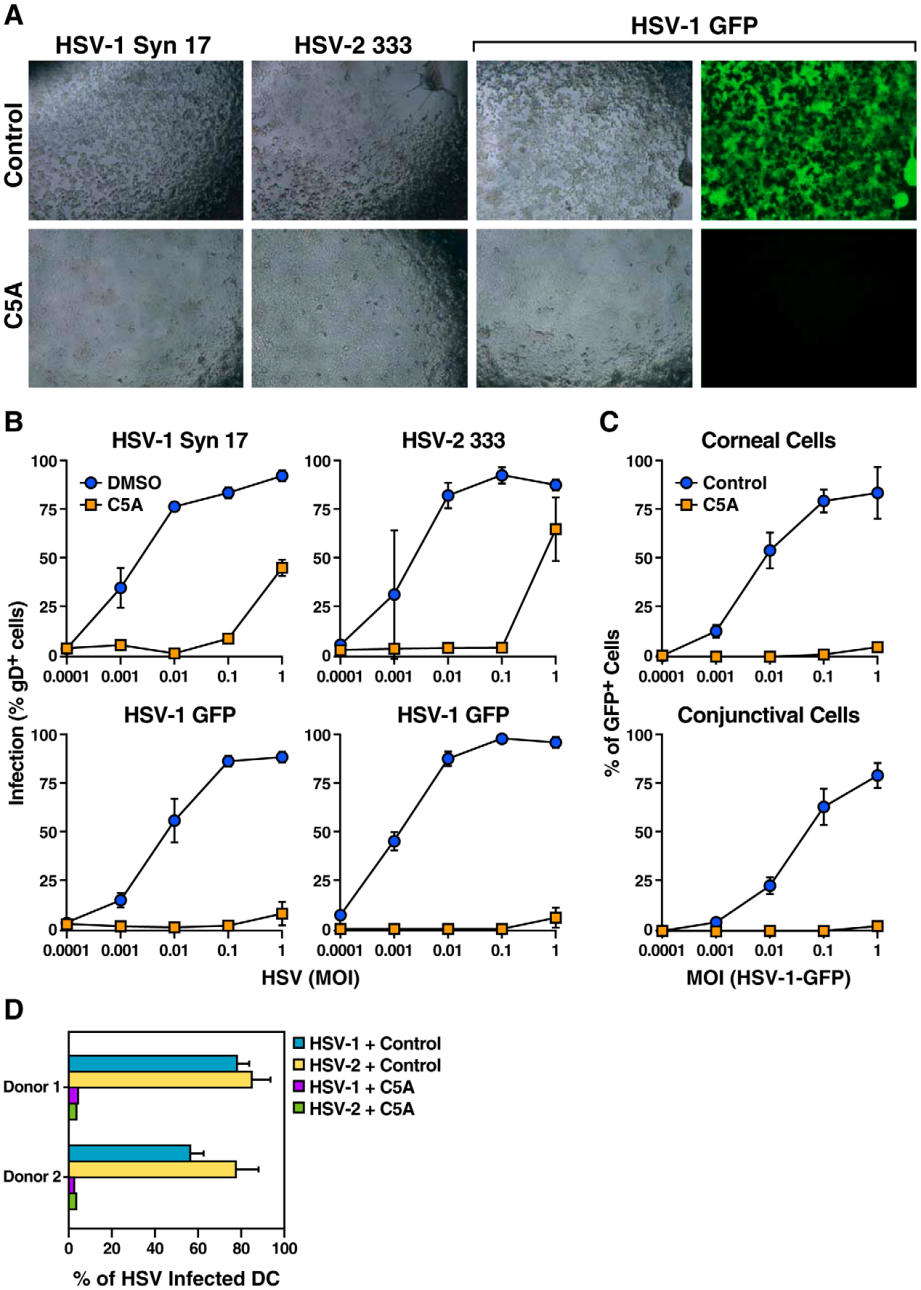
C5A blocks HSV infection. A. Vero cells were seeded in a 24-well plate and infected with increasing concentrations (MOI from 0.0001 to 1) of HSV-1 (Syn 17), HSV-2 (333) or HSV-1-GFP. C5A (5 µM) or control DMSO was added immediately after adding viruses to target cells. Two days post-infection, HSV-infected cultures (MOI 0.1) were analyzed by microscopy and representative pictures are depicted. B. Two days post-infection, infection of Vero cells was quantified by FACS either by mouse anti-HSV gD IgG2a immunostaining (HSV-1 Syn 17 and HSV-2 333) or by GFP content (HSV-1-GFP). For the gD cell surface staining, cells were washed and trypsinized 3 h post-infection to remove the original inoculum, and rabbit FITC-anti-mouse IgG were used to quantify bound mouse anti-HSV gD IgG. C. Human corneal and conjunctival epithelial cells were exposed for two days with increasing concentrations of HSV-1-GFP together with C5A (5 µM) or control DMSO. Infection was scored by GFP content. D. Primary human DC isolated from two donors were incubated with HSV-1 or HSV-2 (MOI of 0.1) together with C5A (5 µM) or control DMSO. HSV infection was quantified by cell surface expression of HSV gD two days post-infection as described above. Error bars represent standard errors of triplicates. Results are representative of two independent experiments.

We investigated whether infection of HSV-1 and -2 could be inhibited by C5A. Vero cells were infected with HSV-1, HSV-1-GFP and HSV-2 in the presence of C5A. C5A (5 µM) or control DMSO was added immediately after adding viruses to target cells. In the absence of C5A, the cell monolayer was destroyed after two days and cells exhibited a round morphology, indicative of HSV infection (Figure 1A, top panels). In contrast, in the presence of C5A, cell monolayers were mostly unaffected, suggesting that C5A prevented HSV infection (Figure 1A, bottom panels). Furthermore, C5A prevented HSV-1 replication in the cell monolayers as observed by decreased in GFP expression in HSV-1-GFP infected cultures in the presence of C5A. (Figure 1A, bottom right panel).

To further measure the efficacy of C5A, Vero cells were infected with increasing concentrations of HSV (MOI from 0.0001 to 1) in the presence or absence of C5A. Infection was quantified either by cell surface expression of HSV gD (Figure 1B, top panels and bottom left panel) or by GFP content (Figure 1B, bottom right panel). Remarkably, C5A blocked both HSV-1 and HSV-2 infection at low and intermediate MOI (MOI from 0.0001 to 0.1) (Figure 1B). Partial inhibition by C5A was observed at very high MOI (MOI of 1) (Figure 1B, top panels). C5A also blocked HSV infection in primary human corneal and conjunctival epithelial cells (Figure 1C).

Next we investigated whether C5A prevents infection in other cells than epithelial cells. Dendritic cells (DC) are productively infected with HSV [10]–[14]. Primary human DC were incubated with HSV-1 or HSV-2 in the presence or absence of C5A. HSV infection was quantified by cell surface expression of HSV gD two days post-infection. Remarkably, C5A strongly reduced HSV infection of DC (Figure 1D). Together, these results demonstrate that C5A blocks HSV infection in different target cells *in vitro*.

### C5A diminishes HSV epidermis infection *ex vivo*

**Figure 2:**
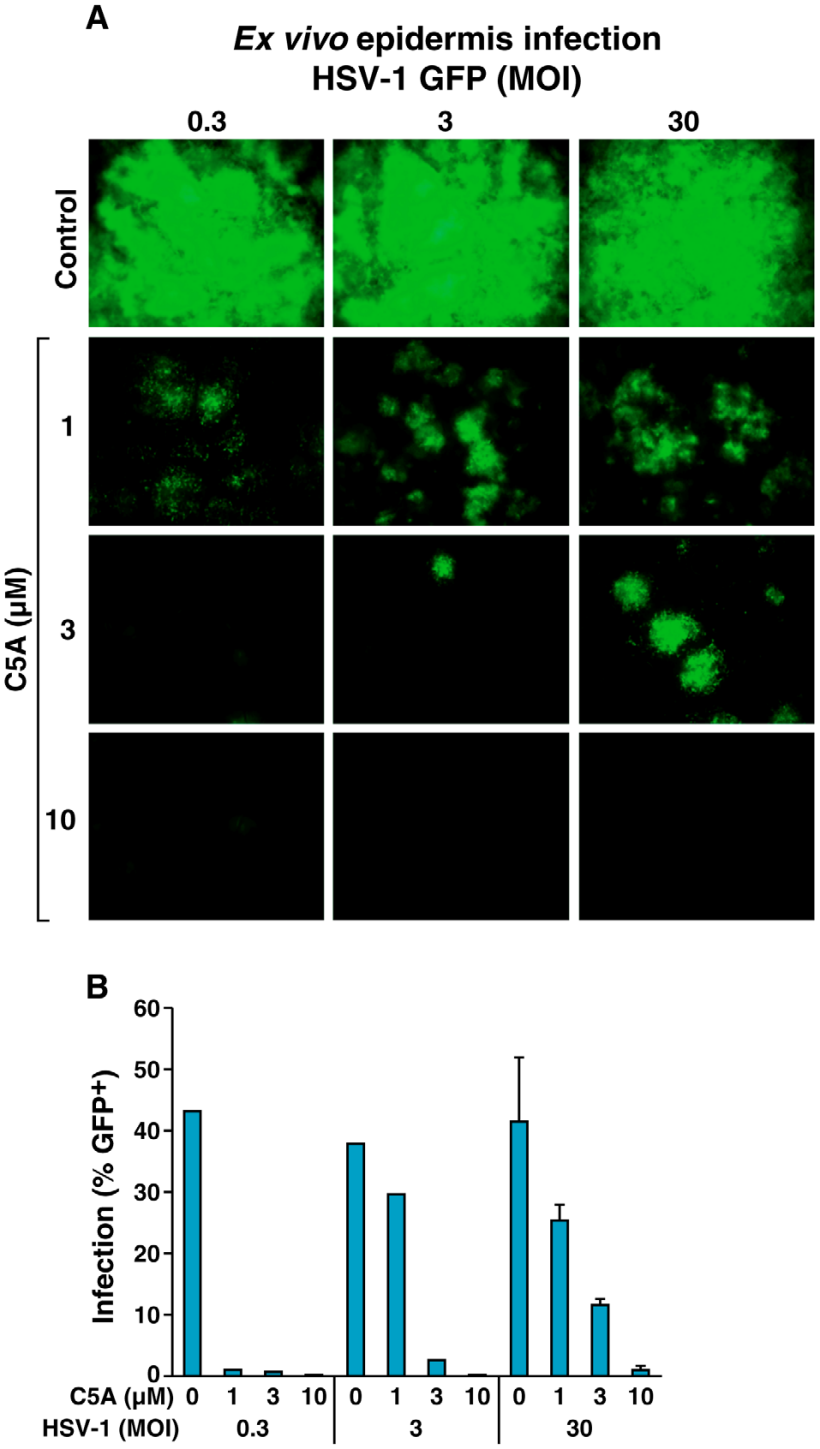
C5A blocks HSV epidermis infection *ex vivo*. A. Epidermal sheets were prepared from human skin and infected with increasing concentrations of HSV-1-GFP (MOI of 0.3, 3 or 30) together with C5A (1, 3 and 10 µM) or control DMSO. Two days post-infection, cell cultures were analyzed by fluorescence microscopy and representative pictures are depicted. B. Epidermal cells were harvested and analyzed for GFP expression by flow cytometry. Error bars represent standard errors of duplicates. These results are representative of 2 independent experiments.

We next investigated the capacity of C5A to interfere with HSV infection *ex vivo*. The primary *in vivo* target cells for HSV are keratinocytes [15]. We therefore infected human epidermal sheets with different concentrations of HSV-1-GFP (MOI of 0.3, 3 and 30). At the moment of infection the sheets were treated with different concentrations of C5A. After 2 days, sheets were analyzed by microscopy and flow cytometry. C5A diminished HSV-1 infection *ex vivo* (Figure 2A and B). The level of inhibition was dependent on the inoculum of the virus (Figure 2A and 2B). Thus, C5A inhibits HSV infection both *in vitro* and *ex vivo*.

### C5A inhibits acyclovir (ACV)- and gancyclovir (GCV)-resistant HSV strains

The drugs of choice for HSV treatment are nucleoside analogues, such as ACV and GCV. Here we examined whether C5A also neutralizes the infectivity of ACV- and GCV-resistant HSV isolates [16]. Specifically, Vero cells were infected with drug-resistant HSV isolates in the presence of increasing concentrations of ACV, GCV or C5A. Infection was quantified by real-time PCR (RT-PCR) as described previously [16] and results expressed as IC_50_ (Table 1). As described previously [16], several of the drug-resistant HSV isolates were resistant to ACV or GCV (Table 1). In contrast, all drug-resistant HSV isolates were sensitive to C5A (IC_50_ from 0.3 to 1 µM) (Table 1). These data demonstrate that C5A can be used to treat drug resistant HSV infections.

**Table 1:** C5A inhibits infectivity of drug-resistant HSV isolates.

*Viruses (HSV)*	*ACV IC_50_*	*GCV IC_50_*	*C5A IC_50_*
97-1716	3.5	3.9	1
95-2723	45.5	>50	0.43
03-16620	12.5	41.5	0.39
95-1552	4.5	0.7	0.5
119681	4.5	0.4	0.75
98-350	0.2	0.2	0.17
03-2787	0.2	0.2	0.3
HSV-1 GFP	ND	ND	0.57

The susceptibility of ACV-, and GCV-resistant HSV isolates was determined by real-time PCR (qPCR) assay. Briefly, Vero cells were inoculated with HSV isolates for 1 h at 37°C. Viral inoculum was removed and cells were incubated, in triplicate, with increasing concentrations of ACV (0.05 to 50 µmol/L), GCV (0.05 to 50 µmol/L) or C5A (0.1 to 50 µM). At 24 h after inoculation, the supernatant was discarded and cells were lysed. Cell lysates were subjected to qPCR as we described previously [16]. Viral load was determined on the basis of a standard curve generated on a stock of HSV-1 strain McIntyre. The IC_50_ (µmol/L) was defined as the concentration of antiviral drug that reduced viral copies by 50%, compared with what was observed for infected control cells to which no drug was added.

### C5A requirements for anti-HSV properties

**Figure 3:**
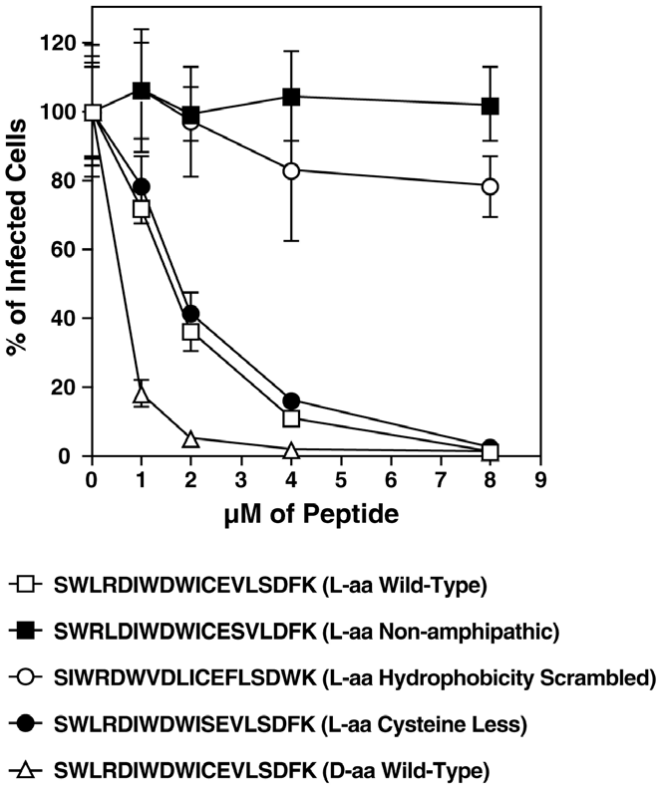
C5A requirements for its anti-HSV effect. Vero cells were seeded in a 24-well plate and infected with HSV-1-GFP (MOI of 0.1). Increasing concentrations (1 to 8 µM) of wild-type or C5A variants were added to cells immediately after virus addition. After 48 h, HSV infection was quantified by FACS by GFP content. Percentage of infection in the absence of peptide was arbitrarily fixed at 100. Error bars represent standard errors of duplicates. Results are representative of two independent experiments.

C5A forms an amphipathic alpha-helical peptide [7]–[8] that might be involved in the anti-viral mechanism. To adress this issue, mutant C5A peptides were generated where an arginine and a serine in SWL**R**DIWDWICEVL**S**DFK wild-type C5A were displaced to create the SW**R**LDIWDWICE**S**VLDFK peptide. Vero cells were infected with HSV and infectivity scored as described above by measuring GFP content (Figure 3). Importantly, these amino acid changes, that disturb the peptide amphipacity, abrogated the anti-HSV effect of C5A (Figure 3). This suggests that the amphipacity of C5A is a prerequirement for the anti-HSV effect. On the other hand, scrambling the hydrophobicity of C5A (S**IW**RD**WV**D**LI**CE**FL**SD**W**K) significantly diminished its anti-HSV activity (Figure 3). Furthermore, putative disulfide bridges formation is not required for the anti-HSV activity of C5A, since the cysteine peptide mutant (SWLRDIWDWI(C)**S**EVLSDFK) exhibits the same anti-viral activity as wild-type C5A (Figure 3). Amino acids are chiral and exist as L or D isomers. Almost all naturally occurring amino acids are L-amino acids and we investigated whether changes in chirality affects the antiviral activity of C5A. Therefore, the anti-HSV efficacy of C5A composed in “conventional” L-amino acids (L-aa) was compared with that of C5A composed in amino acids in D configuration (D-aa). Notably, D-aa C5A was significantly more potent than L-aa C5A in inhibiting HSV infection (Figure 3). Given that proteases recognize mainly L-amino acids, it is likely that the endurance of the D-aa peptide in culture medium (10% FBS) is increased, explaining its enhanced antiviral activity compared to the L-aa peptide. Supporting this hypothesis, the addition of protease inhibitors together with the L-aa peptide significantly increased its antiviral potency (data not shown).

### Mechanisms of anti-HSV action of C5A

**Figure 4:**
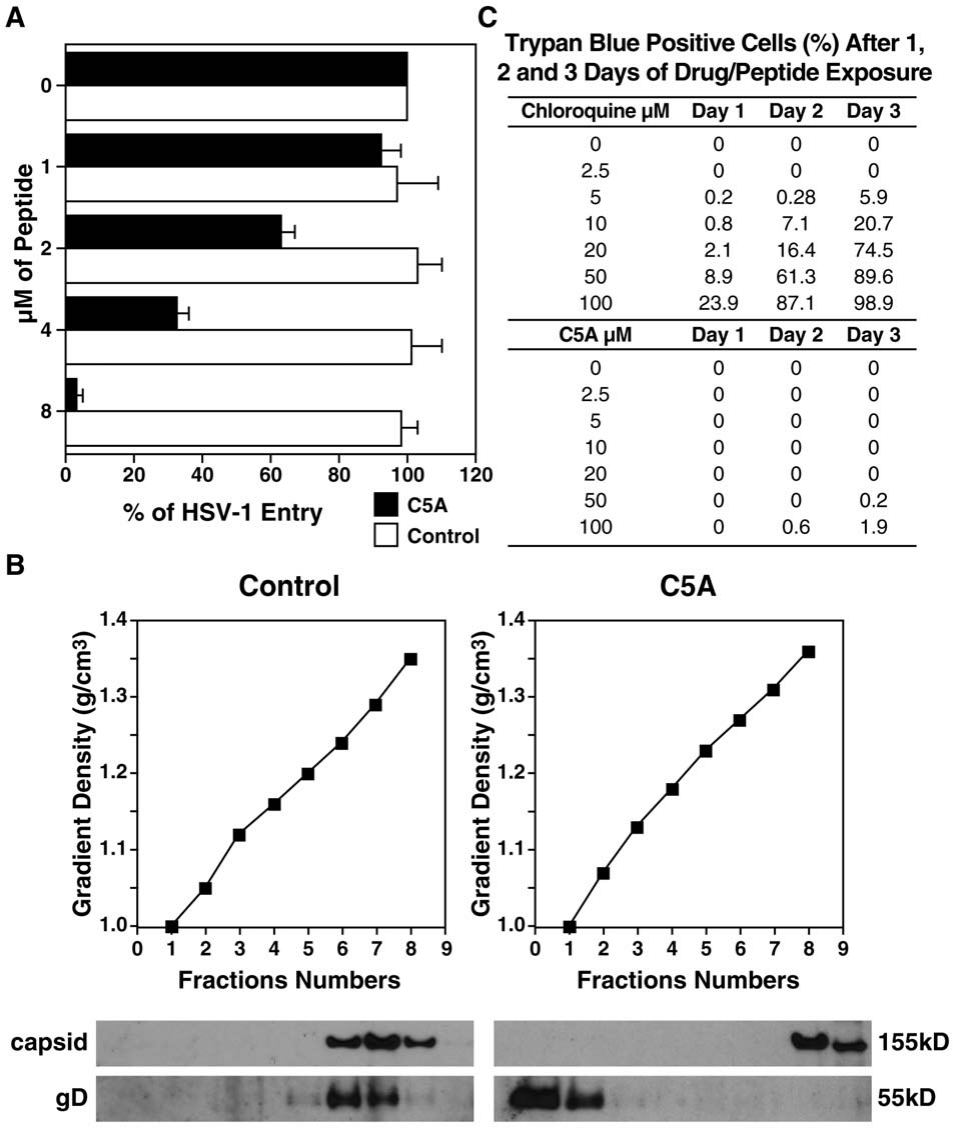
C5A prevents HSV entry by rupturing the integrity of the viral membrane. A. Vero cells were seeded in a 24-well plate and exposed the next day with HSV-1-GFP (MOI of 0.1) for 2 h at 4°C. Increasing concentrations (1 to 8 µM) of wild-type or the control non-amphipathic C5A were then added to cells just prior to shifting the temperature to 37°C. After 2 h, HSV entry was quantified by measuring amounts of HSV capsid internalized. Specifically, cells were washed, trypsinized and lysed. Amounts of HSV capsid in cell lysates were quantified by ELISA. Percentage of entry in the absence of peptide was arbitrarily fixed at 100. Error bars represent standard errors of triplicates. Results are representative of two independent experiments. B. HSV was initially concentrated by loading 20 mL of supernatant of HSV-1-GFP-infected Vero cells on a 20% sucrose cushion. Pelleted viruses were resuspended in 1 mL PBS, incubated with wild-type C5A or the control non-amphipathic C5A variant (5 µM) for 30 min at 37°C and immediately loaded over a 20–70% sucrose density gradient. Each gradient fraction (total of eight fractions) was analyzed for HSV capsid and gD content by immunoblot. The sucrose gradient density of each fraction was determined by measuring the refractive index. Results are representative of 2 independent experiments. C. Human PBMC (1 million) were exposed to increasing concentrations of chloroquine or C5A (0 to 100 µM). At day 1, 2 and 3, the percentage of trypan blue positive was carefully analyzed. Results are representative of 2 independent experiments.

C5A could inhibit HSV infection by interfering during viral entry. To address this issue, we conducted a synchronized assay where Vero cells were exposed to HSV (MOI of 0.1) at 4°C for 2 h and increasing concentrations of C5A or the non-amphipathic control C5A variant were added just prior to shifting the temperature to 37°C (Figure 3). After 2 h, HSV entry was quantified by measuring amounts of HSV capsid internalized. Specifically, cells were washed, trypsinized and lysed. Amounts of internalized virus were quantified by measuring amounts of HSV capsid in cell lysates by ELISA. Importantly, we found that C5A prevented HSV entry in a dose-dependent manner (Figure 4A), suggesting that C5A neutralizes HSV just before or during viral entry.

C5A is virocidal for HIV-1 by destabilizing it at the level of the viral membrane [8]. However, C5A does not influence the infectivity of influenza and vesicular stomatitis (VSV) viruses [7], suggesting that C5A disrupts the envelope integrity of some viruses (HIV-1, HCV), but not others (influenza and VSV). We thus asked whether C5A inhibits HSV, by influencing the integrity of its membrane. Purified HSV virions were resuspended and incubated with wild-type or the non-amphipathic control C5A variant (Figure 3) for 30 min at 37°C and immediately loaded over a 20–70% sucrose density gradient. Each gradient fraction (total of eight fractions) was analyzed for HSV capsid and gD content by Western blotting. The sucrose gradient density of each fraction was determined by measuring the refractive index. Untreated HSV sediments at a density of 1.24 g/cm^3^ as demonstrated by the colocalization of the viral capsid and gD within the gradient (Figure 4B, bottom left panels). Upon C5A treatment, the distribution of capsid and gD within the gradient radically differs. Specifically, gD relocated to the top of the gradient, whereas capsid migrated deeper in the gradient (Figure 4B, bottom right panels). We obtained similar results for gB and gH (data not shown). Altogether these data suggest that C5A partially or completely destabilized the viral membrane, but kept intact the structure of the HSV capsid core. The poor infectivity of C5A-treated HSV virions likely results from the destabilization of their viral membranes.

C5A thus disrupts the membrane of both HSV (Figure 4B) and HIV-1 [8], but preserves the integrity of the cellular plasma membrane [8]. Here, we further verified that C5A exhibits no toxicity onto primary cells. We conducted a trypan blue exclusion assay using human peripheral blood monocytic cells (PBMC) exposed to high concentrations of C5A (2.5 to 100 µM). In contrast to chloroquine, high concentrations of C5A do not exert toxicity on PBMC (Figure 4C), further suggesting that although C5A can disrupt the membrane of viruses, it preserves the integrity of the cellular plasma membrane.

We found that when higher amounts of purified HIV-1 or HSV virions (>100 µg/mL of viral capsid) were used, higher amounts of C5A were required to disrupt viral particles (data not shown). Nevertheless, the ultimate goal of these studies is to use C5A as a microbicidal agent at doses of 100–500 µM, which should suffice to neutralize the expected relatively low amounts of incoming HIV-1 particles during sexual transmission. It is critical to emphasize that we recently demonstrated that the topical application of C5A totally prevented the vaginal transmission of HIV-1 in humanized bone marrow - liver - thymus (BLT) mice [unpublished data]. This is the first proof-of-concept that C5A can be used as a topical microbicidal anti-HIV-1 agent. This also indicates that C5A preserves its antiviral activities in the genital environment. This is in accordance with our observation that *in vitro*, C5A neutralizes HIV-1 infectivity, even diluted in genital fluids (i.e., cervical and vaginal fluids, and seminal plasma) (Table 2). Specifically, seminal plasma was separated from sperm by centrifugation at 500×g for 10 min. The pH was determined before and after mixing with C5A diluted in PBS. We found that the original pH of seminal plasma is approximately 8.0 (n = 8). Fifty µL of serially two-fold diluted peptide in DMEM medium were mixed with 50 µL of JR-CSF and 50 µL of seminal plasma or DMEM. Mixtures were added to TZM reporter cells and infection scored after 48 h. Similar studies were conducted with cervical fluid and vaginal mucus. Importantly, even diluted in seminal plasma or vaginal fluids, C5A blocks HIV-1 infection at a low µM range (IC_50_ from 1 to 5 µM) (Table 2).

**Table 2:** Antiviral efficacy of C5A is preserved in genital fluids.

	Seminal Plasma	Cervical Fluid	Vaginal Mucus
**L-aa C5A**	4.6+/−0.3	4.4+/−0.2	4.8+/−0.3
**L-aa Non-Amphipathic C5A**	>500	>500	>500
**D-aa C5A**	1.4+/−0.1	1.3+/−0.2	1.5+/−0.2
**D-aa Non-Amphipathic C5A**	>500	>500	>500

TZM cells (100,000 cells/mL) were exposed to JR-CSF (1 ng of p24) for 4 h together with increasing concentrations of C5A or control non-amphipathic C5A peptide diluted in various conditions including DMEM (without serum), seminal plasma (8 donors), cervical fluids (4 donors) or vaginal mucus (4 donors). Cells were then washed and infection was measured 48 h after infection by β-galactosidase activity. Data are expressed as IC_50_ in µM. Results are representative of 3 independent experiments.

### HSV neutralization by C5A diminishes HIV-1 transmigration through the genital epithelial barrier

**Figure 5:**
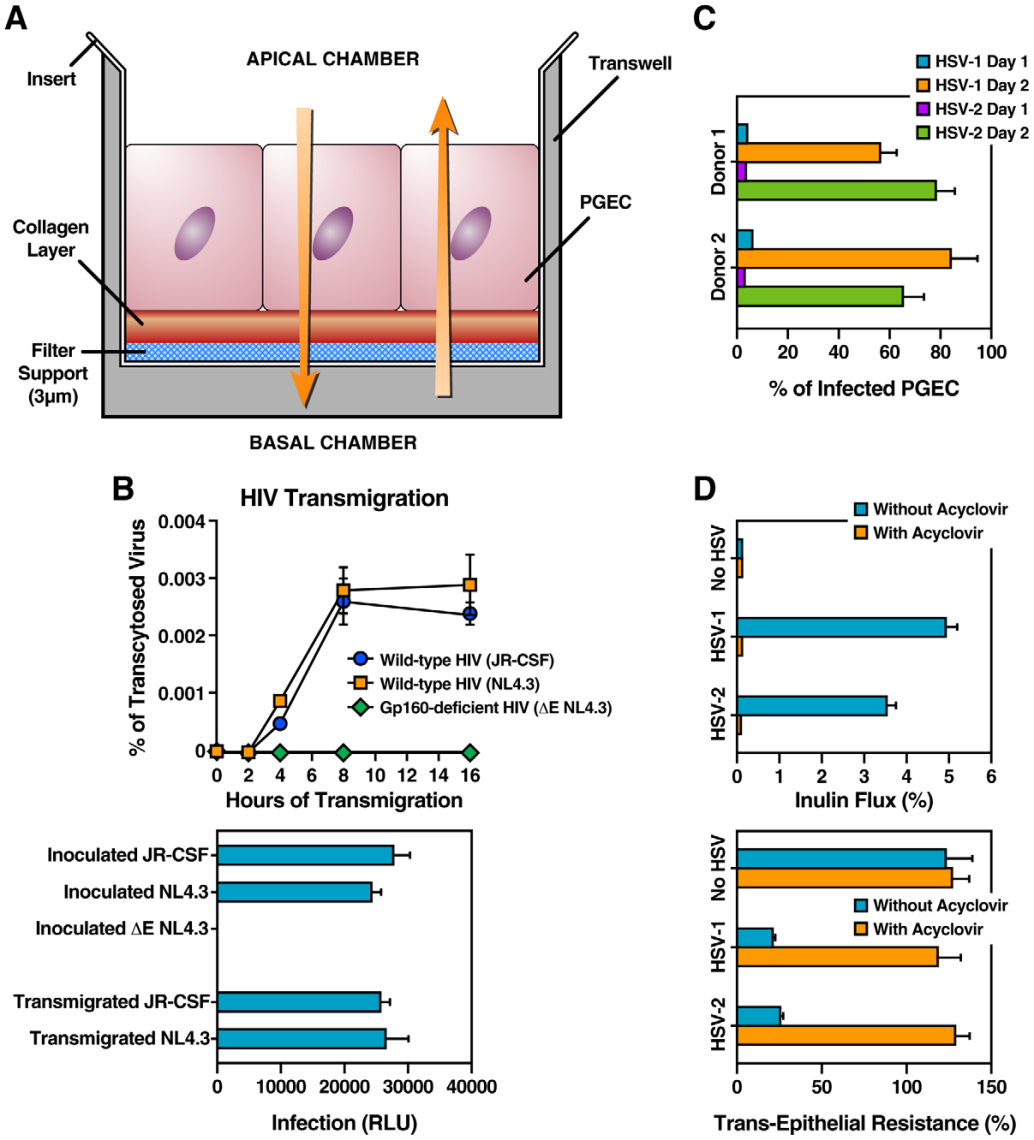
HSV enhances HIV-1 transmigration through the genital epithelial barrier. A. Model for the HIV-1 transwell assay. B. PGEC were seeded onto polycarbonate membrane transwells and cultured until formation of tight junctions was achieved. HIV-1 (wild-type JR-CSF, NL4.3 or gp160-deficient (ΔE) NL4.3) was then added to the apical surface of PGEC and amounts of transcytosed viruses were collected at various time points (2, 4, 8 and 16 h) and quantified by p24 ELISA of the lower chamber corresponding to the basal surface. Data are expressed in percentage of the original viral inoculum (top panel). Infectivity of transcytosed viruses: medium from the basal chamber was collected at different intervals of time, filtered, standardized for p24 content (20 pg), and added to TZM indicator cells. Infection was measured 48 h post-infection by determining levels of beta-galactosidase activity in cell lysates (bottom panel). Results are representative of the results of four independent experiments using PGEC derived from each of the 4 donors. Error bars represent standard deviations. C. PGEC isolated from 2 donors were incubated with HSV-1 or HSV-2 (MOI of 0.005). Two days post-infection, infection of PGEC was quantified by FACS by anti-HSV gD IgG immunostaining. D. PGEC seeded onto the transwells were exposed to HSV-1 or HSV-2 (MOI of 0.005) in the presence or absence of ACV (50 µmol/L). Two days post-infection, the integrity of the PGEC barrier was analyzed by measuring the paracellular passage of the extracellular marker inulin (top panel) as well as the TER of each monolayer (bottom panel) as we described previously [19].

HIV-1 is thought to be sexually transmitted by genital epithelium transmigration. Genital herpes can facilitate HIV-1 acquisition by disrupting the epithelial barrier, thereby increasing exposure of target cells to virus [2], [17]–[18]. We thus investigated whether C5A, by neutralizing HSV, would protect epithelial cells from disruption and therefore slow down HIV-1 transmigration. We previously developed an *in vitro* transwell chamber assay that mimics HIV-1 transcytosis through primary genital epithelial cells (PGEC) [19]. Specifically, PGEC were seeded onto polycarbonate membrane transwells and cultured until formation of tight junctions is achieved. The monolayer on the filter effectively divides the well into an apical compartment and a basolateral compartment (Figure 5A). To ensure the integrity of the PGEC barrier, the elevated transepithelial electrical resistance (TER) of each monolayer was monitored as well as the paracellular passage of the extracellular marker inulin. We found that TER was low at day 1 around 100 Ohm⋅cm^2^, peaked at day 2 up to 400 Ohm⋅cm2 and remained constant for 2 additional days. HIV-1 (1 ng of p24) was added to the apical surface of PGEC in the upper chamber of the transwell system for 8 h at 37°C and the amount of transcytosed virus was quantified by p24 ELISA in the lower chamber medium in contact with the basal PGEC surface [19]. We previously determined that HIV-1 transmigration through PGEC monolayers is maximal after 8 h [19]. Infectivity of transcytosed HIV-1 after 8 h was analyzed using TZM reporter target cells [20]. Wild-type (JR-CSF or NL4.3), but not envelope deficient HIV-1 (ΔE NL4.3), transmigrated through PGEC (Figure 5B, top panel), demonstrating that the PGEC layer does not allow nonspecific transmigration of viruses. Although HIV-1 crosses PGEC as infectious particles (Figure 5B, bottom panel), the efficiency of transcytosis is extremely poor (less than 0.003% of the initial inoculum) (Figure 5B, top panel), suggesting that the genital epithelium serves as a major barrier against HIV-1. We measured 39.8% of viral input present in lower chamber in a control well with no cells so that one could determine how much HIV-1 crosses in the setting of “complete disruption” (data not shown).

After establishing the HIV-1 transmigration assay, we defined conditions that would allow us to examine the enhancing effect of HSV infection of PGEC on HIV-1 transmigration. To address this issue, PGEC monolayers were exposed to a low HSV MOI (0.005). After 2 days, PGEC were washed and exposed this time to HIV-1. HIV-1 transmigration was quantified 8 h post-HIV-1 exposure. We first found that 55–85% of PGEC were infected 2 days post-HSV exposure (Figure 5C). The integrity of the PGEC barrier was monitored before and after HSV exposure. HSV enhanced the passage of FITC-inulin (Figure 5D, top panel) and decreased the TER of the monolayer (Figure 5D, bottom panel), whereas ACV (50 µmol/L)) abolished these effects (Figure 5D). These data confirm that HSV can diminish the impermeability of the PGEC barrier. It is important to note that despite the fact that a significant amount of PGEC (55–85%) were infected with the low HSV MOI (Figure 5C), the integrity of the epithelial barrier was still partially preserved 2 days post-HSV infection (Figure 5D). However, 3–4 days post-HSV infection the PGEC barrier was severely disrupted (data not shown). Moreover, we found that higher MOI (i.e., 0.1 to 1) totally disrupt the integrity of the PGEC barrier already after 2 days (data not shown). Based on these observations, we chose for all subsequent experiments to use a low HSV MOI (0.005) for the initial PGEC infection, and to subsequently expose HSV-infected PGEC to HIV-1 2 days post-HSV infection. We speculated that these conditions mimic physiological conditions, where the integrity of the HSV-infected genital barrier is diminished, but still exists. Interestingly, we observed that PGEC isolated from various donors were constantly more resistant to the lytic effect of HSV than immortalized epithelial cells (i.e., HeLa cells). The origin of this resistance remains to be understood.

**Figure 6:**
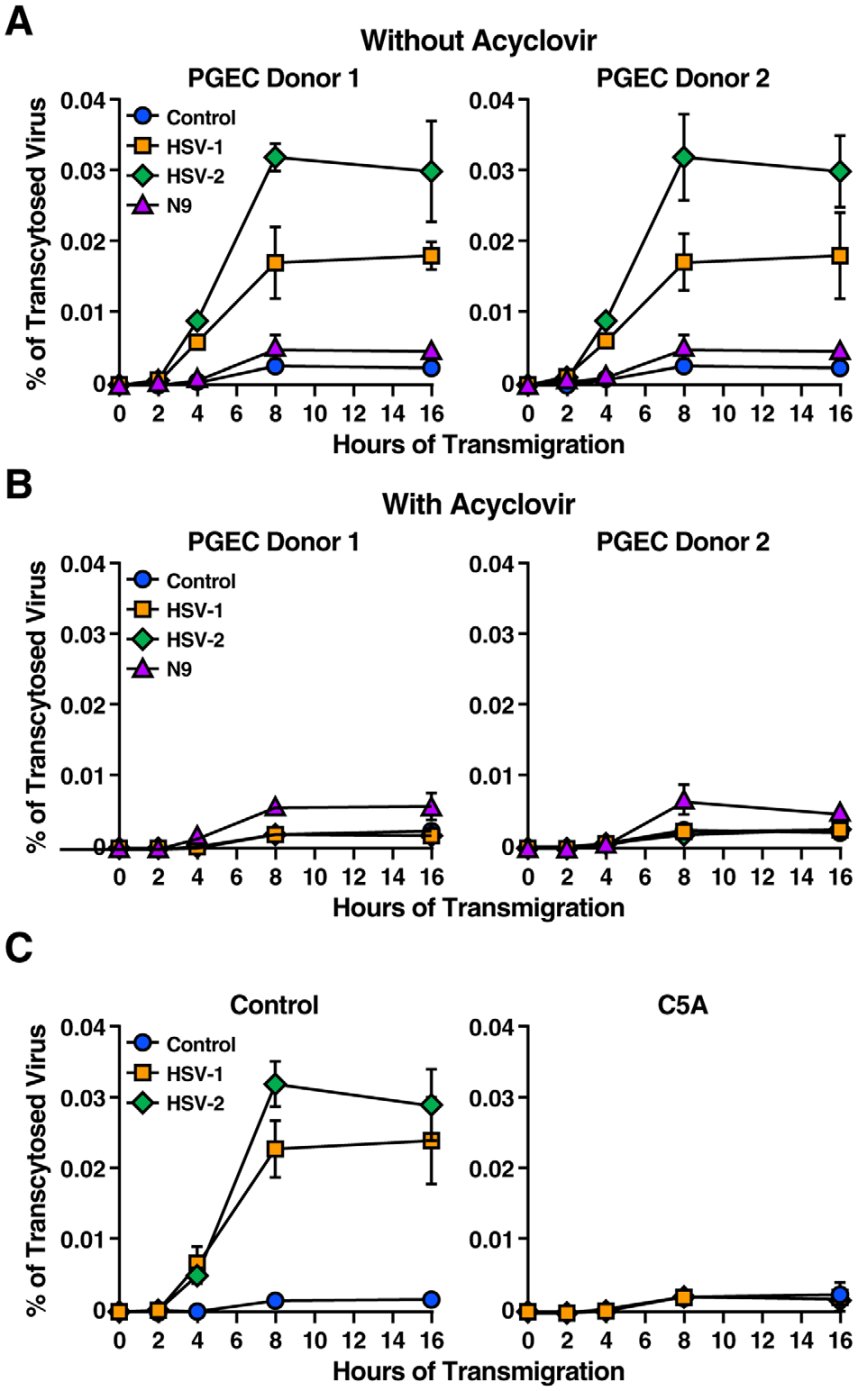
C5A prevents the HSV-mediated disruption of the genital epithelial cell barrier. A. PGEC monolayers were exposed to HSV-1, HSV-2 (MOI of 0.005) or the nonionic detergent N9 (80 µg/mL) for 2 days. HIV-1 (wild-type JR-CSF) (1 ng of p24) was then added to the apical surface of PGEC and amounts of crossed viruses were collected at various time points (2, 4, 8 and 16 h) and quantified by p24 ELISA of the lower chamber corresponding to the basal surface. Data are expressed in percentage of the viral inoculum originally added to the upper chamber. B. Same as A, except that just prior to HSV addition, ACV (50 µmol/L) was added to PGEC monolayers. C. Same as A, except that just prior to HSV addition, wild-type C5A or control non-amphipathic C5A variant (5 µM) was added to PGEC monolayers.

To investigate the effect of HSV infection on HIV-1 transmigration, PGEC monolayers were incubated with HSV-1 or HSV-2 for 2 days, exposed to HIV-1, and amounts of transcytosed HIV-1 were quantified by p24 ELISA in the basal chamber 8 h post-HIV-1 exposure. HSV-1 and HSV-2 infections significantly enhanced HIV-1 crossing (7- and 11-fold, respectively) (Figure 6A). We also analyzed the effect of the nonionic detergent N9, which was originally thought to be spermicide, but which not only failed to offer microbicidal protection, but also increased HIV-1 transmission *in vivo* [6], [21]–[24]. N9 slightly enhanced HIV-1 crossing (Figure 6A), likely due to PGEC cytoxicity as previously described [25]. ACV prevented the HSV-mediated HIV-1 transmigration enhancement (Figure 6B), supporting the notion that HSV infection of PGEC causes the increase of HIV-1 crossing to the lower chamber around disrupted cells.

Next, the antiviral properties of C5A were investigated. PGEC monolayers were first exposed to HSV-1 or HSV-2 together with C5A or the amphipathic control C5A variant for 2 days and washed to remove peptides. HIV-1 was then added to the apical surface of PGEC for 8 h at 37°C and amounts of transcytosed virus were quantified by p24 ELISA in the lower chamber medium. C5A suppressed the HSV-mediated enhancement of HIV-1 transmigration (Figure 6C). These results suggest that C5A, by preventing HSV infection of PGEC (Figure 5C), slows down the disruption of the integrity of the monolayer (Figure 5D), which favors HIV-1 transcytosis (Figure 6A and 6B). In conclusion, C5A, by neutralizing HSV, slows down severely (5–10-fold) the passage of HIV-1 through the genital epithelial barrier.

## Discussion

In this study, we examined the possibility that C5A represents a multipurpose microbicidal candidate that neutralizes both HIV-1 and HSV. Our data indicate that C5A effectively inhibits both HSV-1 and HSV-2 infection of epithelial cells *in vitro* and *ex vivo* by destabilizing the viral membrane without affecting cellular membranes. HSV infection enhances HIV-1 transmission by affecting the integrity of epithelial layers. Notably, our data demonstrate that C5A prevents HSV-induced HIV-1 transmission by preventing HSV replication and thereby disruption of the epithelial layer in an *in vitro* HIV-1 transmigration assay. These data strongly suggest that C5A can be used in strategies to prevent HSV infection and HSV-induced HIV-1 transmission.

A truly safe and effective microbicide is likely to require a combination of drugs that target different steps in the HIV-1 life cycle and provide protection against other STIs known to facilitate HIV-1 infection such as HSV. Epidemiological studies consistently demonstrate a strong link between HSV-2 infection and the risk for HIV-1 acquisition and transmission [1]–[3]. The prevalence of HSV-2 infection among Africans with HIV-1 ranges from 50 to 90%. Asymptomatic shedding is common and is associated with both a higher frequency and a larger amount of HIV-1 in genital secretions. In the present study, we examined the possibility that C5A neutralizes HSV. We found that C5A, at nM-µM range, efficiently inhibits HSV-1 and HSV-2 infection *in vitro*. This is not only true for immortalized cells such as Vero, corneal and conjunctival epithelial cells, but also for primary cells such as human DC and PGEC. Moreover, we showed that C5A blocks HSV epidermis infection *ex vivo*. Thus, C5A neutralizes both HIV-1 and HSV. Importantly, this dual inhibitory effect of C5A is reminiscent of the result of two recent studies, which showed that PPCM and cyclovir ProTides represent a new class of antivirals that suppress both HIV-1 and HSV infection [26]–[27]. We are aware that a high nM to low µM range inhibitory effect is not ideal for a microbicidal candidate and that the activity of C5A will be coital dependent. However, it is important to emphasize that we are currently synthesizing a second generation of peptides using C5A archetype and are testing them for antiviral activities in genital fluids. Our goal is to identify a peptide, which is active at a low nM range in genital fluids.

We previously examined the possibility that C5A prevented HIV-1 transcytosis because it is toxic for PGEC [8]. PGEC were exposed twice daily to high concentrations (200 µM) of C5A for a week. To maintain a continuous exposure of cells to the peptide, no washes were performed. Cell viability was evaluated by methyl thiazol tetrazolium (MTT)-based colorimetric assessment. As a control, cells were exposed to the detergent saponin. In contrast to 0.01% saponin, we found that C5A applied to cells at a concentration of 10- to 100-fold greater than that which blocks HIV-1 infection is not toxic to PGEC [8]. The fact that the peptide disrupts the HIV-1 membrane without PGEC toxicity, suggested that C5A prevents HIV-1 transmigration without interfering with epithelial integrity [8]. This is in accordance with our observation that C5A does not affect the cellular viability of the epidermal explants (data not shown).

It remains to be understood why the integrity of the cellular membrane is preserved, but not that of the viral membrane (i.e., HIV-1 and HSV). One simple possibility is that the viral membrane is more fragile than the cell membrane. In this scenario, the C5A-mediated destabilization of the membrane would have a more dramatic effect on a viral than a cell membrane. Another possibility is that the viral membrane is enriched with a C5A ligand. In this scenario, a denser concentration of C5A binding sites would pre-exist in the viral membrane than in the cell membrane. Further work is required to determine whether or not C5A can disrupt the integrity of all enveloped viruses, and why C5A does not affect the cell membrane even when used at high concentrations [8]. Nevertheless, the goal of this study is to determine whether C5A is a multipurpose anti-HIV-1 microbicidal agent, which can dually neutralize HIV-1 and HSV, and prevents HIV-1 transmission by inhibiting multiple steps of HIV-1 transmigration through the genital barrier.

We obtained evidence that treating HSV with C5A disturbs the integrity of the viral membrane. This is consistent with our previous observation that C5A disrupts the integrity of HIV-1 [8]. It is likely that the release of the viral glycoproteins – gp120 for HIV-1 [8] and gD/B/H for HSV (this study) – results from the disruption of the integrity of the viral membrane. It is important to note that C5A corresponds to a small N-terminal region (amino acid 3–20) of the HCV NS5A (477 amino acids) [7]. The sequence of C5A encompasses the region responsible for the anchoring of NS5A into the ER membrane [9]. Thus, one can envision that the anchoring of multiple C5A molecules into the HIV-1 or HSV membrane somehow distabilizes the integrity of the fragile viral membrane, resulting in the release of components of the viral membrane. We previously showed that pre-treating HIV-1 with proteases does not abrogate the C5A-mediated HIV-1 rupture [8], suggesting that the membrane-associated C5A ligand is not proteinous. Further work is required to identify the C5A ligand (i.e., a lipid) within the membrane HIV-1 and HSV. The C5A ligand could represent a novel target for the development of anti-HIV-1 microbicides.

Interestingly, C5A has a different impact on capsid cores of HIV-1 and HSV. For HIV-1, C5A completely disrupted the integrity of the capsid core reflected by the fact that all HIV-1 capsid proteins relocated to the top of the sucrose gradient upon HIV-1 exposure to C5A [8]. We postulated that C5A disrupts the integrity of HIV-1 capsid cores by destabilizing the physical linkage between the mature conical capsid core and the viral envelope [8]. In contrast, in this study we found that HSV capsid proteins, rather than relocating to the top of the gradient after HSV treatment with C5A, migrated deeper in the gradient, suggesting that HSV capsid cores remain intact after C5A treatment, but that the partial or complete removal of the lipidic viral membrane by C5A, render them denser, explaining their deepened migration in the gradient. These data suggest that the HIV-1 capsid core is more fragile than the HSV capsid core after viral membrane removal by C5A. Nevertheless, it is likely that the C5A removal of the HIV-1 and HSV glycoproteins responsible for viral entry suffices to neutralize the infectivity of these pathogens.

In this study, we used an *in vitro* transwell chamber assay that mimics HIV-1 transcytosis through PGEC. We showed that HIV-1 infectious particles transmigrate through PGEC in a gp160-dependent manner. As we previously reported, the efficiency of cell-free HIV-1 transcytosis is extremely poor [8], suggesting that the genital epithelium serves as a major barrier against HIV-1 transmission. We confirmed that HSV infects PGEC and damages the impermeability of the PGEC monolayer. The HSV infection of PGEC significantly enhances HIV-1 transcytosis. It is important to note that we found that the disruption of the PGEC barrier by HSV is highly dependent on both the viral MOI and the length of infection. Specifically, a high HSV MOI (0.1 to 1) totally disrupted the PGEC barrier already 2 days post-infection. Moreover, a low HSV MOI (0.005), as used in this study, greatly damaged the PGEC barrier 4 to 5 days post-HSV infection. Surprisingly, PGEC were constantly more resistant to the lytic effect of HSV than immortalized genital epithelial cells. As mentioned above, the enhanced endurance of PGEC to HSV infection remains to be elucidated.

We found that C5A abolished the HSV-mediated enhancement of HIV-1 transmigration, suggesting that C5A, by preventing HSV infection of genital epithelial cells, preserves the integrity of the PGEC monolayer. By demonstrating that C5A blocks HIV-1 transmission as well as HSV infection, especially with ACV-resistant HSV strains, this study further suggests that C5A represents an attractive microbicidal candidate. Supporting this notion, we recently demonstrated that a topical application of C5A offers complete protection against a vaginal challenge of HIV-1 in a humanized BLT mouse transmission model [unpublished data]. It is important to note that a recent study indicates that C5A possesses chemo-attractant properties [28]. If this is true, this property may represent a concern for the use of C5A as microbicide. For this reason, we are currently screening a second generation of C5A derivates for their chemo-attractant properties. Our goal is to identify a peptide with potent anti-HIV-1 activities, but deprived of chemo-attractant properties.

In conclusion, C5A represents a multipurpose microbicide candidate, which neutralizes both HIV-1 and HSV, and interferes with mechanisms thought to be critical for HIV-1 sexual transmission such as HIV-1 transcytosis and HSV-mediated disruption of the integrity of the genital epithelial barrier. Development of a topical microbicide that targets both HIV-1 and HSV such as C5A may prove a powerful strategy for reducing HIV-1 as epidemiological studies consistently demonstrate synergy between these two pathogens.

## Materials and Methods

### Viruses

HSV-1 Syn17+, HSV-1 VP16-GFP [5], and HSV-2 333 were grown and titrated on Vero cells. ACV- and GCV-resistant HSV isolates were obtained from G. Verjans. HIV-1 (wild-type JR-CSF, NL4.3 or gp160-deficient NL4.3) viruses were produced by transfection of 293T cells with JR-CSF, pNL4.3 or gp160-deficient pNL4.3 ΔE proviral plasmids (obtained respectively from I. Chen, M. Martin and N. Landau through the NIH AIDS Research and Reference Reagent Program).

### Cells

TZM cells (obtained from J. C. Kappes, X. Wu, and Tranzyme Inc. through the NIH AIDS Research and Reference Reagent Program). TZM cells express CD4, CXCR4, and CCR5, which render them susceptible to infection, and contain an integrated *Escherichia coli lacZ* gene driven by the HIV-1 long terminal repeat [20]. Upon infection, Tat production from the integrated provirus leads to activation of the *lacZ* reporter, resulting in synthesis of beta-galactosidase in these cells. Infected cells are identified by enzymatic activity measurement 48 h post-infection, allowing quantitation after a single round of infection as described previously [20]. PGEC were provided by B. Kahn of the Department of Obstetrics and Gynecology at the Scripps Clinic. By rotation of cotton swabs against the vaginal walls, several million cells were collected per individual. Cells were immediately placed in sterile phosphate-buffered saline (PBS), held at 4°C, and transported to the laboratory. After centrifugation (300× *g* for 5 min), the cell pellet was digested in 1 mg/mL of collagenase-dispase (Roche Molecular Biochemicals) containing 1 mg/mL of DNase (Sigma) and 0.15 mg/ml of Na-*p*-tosyl-L-lysine chloromethyl ketone (Sigma) for 1 h at 37°C. The digest was spun down (1,000× *g* for 20 min) and resuspended in 250 mg/mL of PBS-bovine serum albumin (PBS-BSA). After additional centrifugation, the pellet was resuspended in 5 mg/ml of PBS-BSA and loaded onto a 50% Percoll gradient. PGECs were then isolated from contaminating cells by fluorescence-activated cell sorting (FACS) as previously described [19] and propagated into collagen type I-coated T-25 flasks in Dulbecco's modified Eagle medium F12 medium containing 10% fetal calf serum and epithelial cell growth supplement (100 µg/mL) (Sigma). We obtained ethics approval from the Scripps IRB/Committee for the Protection of Human Subjects approval for PGEC collections and at the Scripps hospital where participants were recruited and human experimentation was conducted. We obtained written informed consent from all participants involved in the study. As a second source of PGEC, cells were obtained from Whittaker (customized request). PGEC were passaged fewer than three times prior to use in order to maintain their original features. Vero and 293T cells were generously obtained from C. Aiken, whereas human corneal and conjunctival epithelial cell lines were provided by I. Gipson and G. Verjans. Primary human DC were purified and characterized as described previously [8].

### *In vitro* and *ex vivo* infections

Cells (Vero, DC, corneal and conjunctival epithelial cells) were seeded into a 24-well plate and infected with different concentrations of HSV (HSV-1, -2 and HSV-1-GFP) together with C5A or the DMSO control. C5A peptides were dissolved in DMSO and subsequently diluted in RPMI or DMEM in the absence of serum. Forty-eight hours post-infection, cells were harvested and stained with antibodies against HSV glycoprotein gD (T111; Novus biologicals, Littleton, CO, USA) for HSV-1 and -2 infection or analyzed by flow cytometry for HSV-1-GFP infection. Cells were washed and fixed with 4% PFA/PBS and analyzed by flow cytometry. In some cases, cell morphology and GFP expression were analyzed using a Leica DMIL fluorescence microscope (Leica Microsystems Wetzlar, Germany) and pictures were taken using a Leica DFC 320 camera (Leico Microsystems). Epidermal sheets were prepared as we previously described [29]. The sheets were infected with different concentrations of HSV-1-GFP by adding the virus underneath the sheets into the medium. Different concentrations of C5A or the appropriate volume of the DMSO control solution was added in a total volume of 500 µL. After 2 h, 1.5 mL complete medium was added and the sheets were cultured for 2 days. The sheets were analyzed by fluorescence microscopy or by measuring GFP expression by flow cytometry. The susceptibility of ACV- and GCV-resistant HSV isolates was determined by real-time PCR (qPCR) assay as described previously [16]. Briefly, Vero cells were inoculated with HSV-1 for 1 h at 37°C. Viral inoculum was removed and cells were incubated, in triplicate, with different concentrations of ACV, GCV or C5A. Twenty-four hours after inoculation, the supernatant was discarded and cells were treated with 300 µL of lysis buffer (2.5 mmol/L MgCl_2_, 50 mmol/L KCl, 10 mmol/L Tris-HCl, 0.45% Tween 20, 0.45% Nonidet-P40, and 200 µg/mL proteinase K) and were incubated for 90 min at 56°C. A 10 µL portion of the 10-fold diluted lysate was subjected to qPCR by use of an Applied Biosystems 7000 Sequence Detection system as described previously [16]. Viral load was determined on the basis of a standard curve generated on a stock of HSV-1 strain McIntyre counted by electron microscopy (Advanced Biotechnologies), and the HSV-1 isolates were assayed at least 3 times. The IC_50_ was defined as the concentration of antiviral drug that reduced viral copies by 50% compared with what was observed for control infected cells to which no antiviral drug was added.

### HSV entry assay

Vero cells (100,000 cells) were seeded in a 24-well plate and exposed to HSV-1-GFP (MOI of 0.1) at 4°C for 2 h. Increasing concentrations (1 to 8 µM) of C5A peptides were then added to cells just prior to shifting the temperature to 37°C. After 2 h, cells were extensively washed, trypsinized and lysed. HSV entry was quantified by measuring amounts of HSV capsid in cell lysates by ELISA. Recombinant HSV capsid and anti-HSV capsid 3B6 monoclonal and NC1 polyclonal antibodies were used to establish the HSV capsid sandwich ELISA.

### Virus sedimentation assay

HSV was initially concentrated by loading 20 mL of supernatant of HSV-1-GFP-infected Vero cells on a 20% sucrose cushion. Pelleted viruses were resuspended in 1 mL PBS, incubated with wild-type C5A or control non-amphipathic C5A for 30 min at 37°C and immediately loaded over a 20–70% sucrose density gradient. After ultracentrifugation at 20,000 rpm for 24 h in a SW-41 T rotor, fractions (1 mL) were collected and tested for their content of viral proteins. Each gradient fraction was analyzed for HSV capsid and gD content by immunoblot. The sucrose gradient density of each fraction was determined by measuring the refractive index.

### HIV-1 transmigration assay

PGEC were seeded onto the upper face of collagen I and fibronectin-coated 12-mm-diameter, 3-µm-pore-size polycarbonate membrane transwells at a density of 10^5^ cells/well and were cultured until formation of tight junctions was achieved. The inserts were fed every two days. The monolayer on the filter effectively divides the well into an apical compartment and a basolateral compartment. To ensure the integrity of the PGEC barrier, we monitored the elevated transepithelial electrical resistance (TER) of each cell monolayer as we described previously [19] using a Millicell-ERS Electrical Resistance System (Millipore, Bedford, MA). The resistance of the polycarbonate membrane in transwells was subtracted from all readings. To further monitor the integrity of the PGEC barrier, we also measured the paracellular passage of the extracellular marker inulin by determination of the permeability coefficient as evaluated by a diffusion assay using inulin as described previously [19]. Transwells with PGEC monolayers were incubated under different experimental conditions in the presence of FITC-inulin (0.5 mg/mL) in the upper well. At varying times during the experiment, 50 µL of apical and basal media was withdrawn and the fluorescence was measured using a fluorescence plate reader (BioTEK Instruments, Winooski, VT). The flux into the apical well was calculated as the percentage of total fluorescence administered into the basal well per cm^2^ surface area. The integrity of the PGEC monolayers was analyzed before and after HSV exposure. After analyzing the integrity of the monolayer on the transwell filters, PGEC were exposed to HIV-1 (added to the upper chamber) and release of HIV-1 into the basal chamber was monitored by measuring amounts of particulate capsid in the basal chamber by p24 ELISA. Infectivity of transcytosed HIV-1 particles was scored on TZM reporter cells as described above.
